# Azacitidine in 302 patients with WHO-defined acute myeloid leukemia: results from the Austrian Azacitidine Registry of the AGMT-Study Group

**DOI:** 10.1007/s00277-014-2126-9

**Published:** 2014-06-21

**Authors:** Lisa Pleyer, Sonja Burgstaller, Michael Girschikofsky, Werner Linkesch, Reinhard Stauder, Michael Pfeilstocker, Martin Schreder, Christoph Tinchon, Thamer Sliwa, Alois Lang, Wolfgang R. Sperr, Peter Krippl, Dietmar Geissler, Daniela Voskova, Konstantin Schlick, Josef Thaler, Sigrid Machherndl-Spandl, Georg Theiler, Otto Eckmüllner, Richard Greil

**Affiliations:** 1 3rd Medical Department with Hematology and Medical Oncology, Hemostaseology, Rheumatology and Infectious Diseases, Laboratory for Immunological and Molecular Cancer Research, Oncologic Center, Paracelsus Medical University Hospital Salzburg, and Center for Clinical Cancer and Immunology Trials at Salzburg Cancer Research Institute, Müllner Hauptstrasse 48, 5020 Salzburg, Austria; 2 Department for Internal Medicine IV, Hospital Wels-Grieskirchen, Wels, Austria; 3 1st Medical Department with Hematology, Stem Cell Transplantation, Hemostatsis and Medical Oncology, Elisabethinen Hospital, Linz, Austria; 4 Department of Hematology, Medical University, Graz, Austria; 5 Internal Medicine V (Hematology and Oncology), Innsbruck Medical University, Innsbruck, Austria; 6 Third Medical Department, Hanusch Hospital, Vienna, Austria; 7 First Department of Internal Medicine, Center for Oncology and Hematology, Wilhelminenspital, Vienna, Austria; 8 Department for Hematology and Oncology, LKH Leoben-Eisenerz, Leoben, Austria; 9 5th Medical Department with Oncology und Palliative Medicine, Hietzing, Vienna, Austria; 10 Internal Medicine, Hospital Feldkirch, Feldkirch, Austria; 11 Division of Hematology and Hemostaseology, Department of Internal Medicine I, Medical University of Vienna, Vienna, Austria; 12 Department for Internal Medicine, LKH Fuerstenfeld, Fuerstenfeld, Austria; 13 Department for Internal Medicine, with Nephrology, Gastroenterology and Hepatology, Hematology and Medical Onkology, Intensive Care Unit, and Rheumatology, Klinikum Klagenfurt am Wörtersee, Pörtschach am Wörthersee, Austria; 14 Internal Medicine 3, Center for Hematology and Medical Oncology, General Hospital-Linz GesmbH, Linz, Austria; 15 Institut für Waldwachstumsforschung, Universität für Bodenkultur, Vienna, Austria

**Keywords:** Austrian Azacitidine Registry, Azacitidine, AML, Overall survival, Prognostic factors, Bone marrow blasts

## Abstract

Data on efficacy and safety of azacitidine in acute myeloid leukemia (AML) with >30 % bone marrow (BM) blasts are limited, and the drug can only be used off-label in these patients. We previously reported on the efficacy and safety of azacitidine in 155 AML patients treated within the Austrian Azacitidine Registry (clinicaltrials.gov identifier NCT01595295). We herein update this report with a population almost twice as large (*n* = 302). This cohort included 172 patients with >30 % BM blasts; 93 % would have been excluded from the pivotal AZA-001 trial (which led to European Medicines Agency (EMA) approval of azacitidine for high-risk myelodysplastic syndromes (MDS) and AML with 20–30 % BM blasts). Despite this much more unfavorable profile, results are encouraging: overall response rate was 48 % in the total cohort and 72 % in patients evaluable according to MDS-IWG-2006 response criteria, respectively. Median OS was 9.6 (95 % CI 8.53–10.7) months. A clinically relevant OS benefit was observed with any form of disease stabilization (marrow stable disease (8.1 months), hematologic improvement (HI) (9.7 months), or the combination thereof (18.9 months)), as compared to patients without response and/or without disease stabilization (3.2 months). Age, white blood cell count, and BM blast count at start of therapy did not influence OS. The baseline factors LDH >225 U/l, ECOG ≥2, comorbidities ≥3, monosomal karyotype, and prior disease-modifying drugs, as well as the response-related factors hematologic improvement and further deepening of response after first response, were significant independent predictors of OS in multivariate analysis. Azacitidine seems effective in WHO-AML, including patients with >30 % BM blasts (currently off-label use). Although currently not regarded as standard form of response assessment in AML, disease stabilization and/or HI should be considered sufficient response to continue treatment with azacitidine.

**Electronic supplementary material:**

The online version of this article (doi:10.1007/s00277-014-2126-9) contains supplementary material, which is available to authorized users.

## Introduction

Acute myeloid leukemia (AML) is an aggressive disease with an unfavorable prognosis [1, 2]. Treatment with curative potential, i.e., conventional chemotherapy and/or allogeneic stem cell transplantation, is rarely an option for elderly patients due to high age, comorbidities, poor performance status, and/or adverse cytogenetics. Azacitidine is approved for AML with 20–30 % bone marrow (BM) blasts. Approval was based on the pivotal AZA-001 trial [3, 4]. Currently, the 001-follow-up trial (clinicaltrials.gov identifier NCT01074047) is underway with the same design, but limited to patients with >30 % BM blasts, with the aim to widen the indication of azacitidine to AML patients as defined by WHO, i.e., irrespective of BM blast count. Until the results of this trial become available, treatment of AML patients with more than 30 % BM blasts with azacitidine remains an off-label use.

We previously reported on the use, efficacy, and safety of azacitidine in 155 WHO-AML patients treated in a real-life setting. We herein update this report with a population almost twice as large (*n* = 302). The aim of the current publication was to potentially confirm, consolidate, and validate our previous results. The primary endpoint was evaluation of efficacy (i.e., response) of azacitidine in patients with AML defined according to WHO criteria (including patients with >30 % BM blasts). The secondary endpoints were safety (i.e., toxicity and adverse events), overall survival (OS), and statistical analysis of factors known or thought to influence overall survival in order to establish prognostic markers (Table 1 [4–14]).

**Table 1:** Comparison of all current full publications on AML patients treated with azacitidine

Variable	No. of AML patients	Inhabitants (mio)	No. of AML patients treated with AZA/capita	Phase	Median age (range)	AZA schedule	AZA dose	Median cycles (range)	Median FU months	Median OS months	ORR (ITT), % CR/(m)CR, PR, HI	BM blasts *n* patients ≤30 % >30 %	Cytogenetics, %
CALGB 8421	25	311.6	0.08	I/II-subanal.	65 (33–82)	d1–7	75 mg/m^2^	ND	ND	ND	48 12, 4, 32	WHO-AML ND	ND
CALBG 8921	26	311.6	0.08	II-subanal.	66 (23–82)	d1–7	75 mg/m^2^	ND	ND	ND	35 12, 0, 23	WHO-AML ND	ND
CALG 9221	27	311.6	0.09	III-subanal.	69 (31–92)	d1–7	75 mg/m^2^	ND	ND	19.3	37 7, 0, 30	WHO-AML ND	ND
Germany	40	81.7	0.49	I/II	72 (46–87)	d1–5	75 mg/m^2^	3 (0–16)	13	3	30 5, 8, 18	17 ND	Int: 70^a^ High: 30
AZA 001	55	897.3^b^	0.06	III-subanal.	70 (52–80)	d1–7	75 mg/m^2^	8 (1–39)	20	24.5	ND 18, ND, ND	13 0	Int: 69^c^ High: 26
Pennsylvania	20	12.7	1.57	Retrosp.	69 (44–80)	d1–7	75 mg/m^2^	ND	ND	2.5–15^d^	60 20, 25, 15	ND 6	Int: 60^e^ High: 20
France	26	65.4	0.40	Retrosp.	69 (37–89)	d1–7	75 mg/m^2^	6 (1–28)	20	8	≥39 16, 23, ND	MPD-AML 17	Int: 38^f^ High: 46
Holland	31	16.7	1.86	Retrosp.	70 (40–84)	d1–7	75 mg/m^2^	5 (1–19)	8	6–16^d^	39 23, 3, 13	WHO-AML ND	Int: 68^c^ High: 32
Lausanne	38^g^	2.0^h^	19	Retrosp.	68 (25–86)	d1–5	100 mg/m^2^	6 (3–20)	12.2	8.6	≥31 13, 4, 31	9 29	Int: 64^i, g^ High: 29
Holland	55	16.7	3.29	Retrosp.	73 (59–84)	d1–7	75 mg/m^2^	6 (1–27)	ND	12.3	42 31, 11, 7	38 17	Int: 64^a^ High: 33
Italy	82	60.6	1.36	Retrosp.	77 (46–87)	d1–7	60 %, 75 mg/m^2^ 40 %, 100 mg flat	4 (1–22)	12	7–9^d^	36 20, 12, 34	33 49	Int: 37^a^ High: 23
France	149	65.4	2.28	Retrosp.	74 (31–91)	d1–5 (18 %) d1–7 (78 %) others (4 %)	85 %, 75 mg/m^2^ 15 %, 100 mg flat 41 %, +ATRA ± VPA 22 %, +HU	5 (1–31)	ND	9.4	28 23, 5, ND	62 87	Int: 53^a^ High: 40
Austria	155	8.2	18.9	Retrosp.	73 (33–91)	d1–5 (16 %) d1–7 (57 %) 5-2-2 (22 %) others (5 %)	78 %, 75 mg/m^2^ 22 %, 100 mg flat	4 (1–24)	7.7	9.8	45 13, 21, 9	57 98	Int: 74^a^ High: 17
Present study	302	8.2	36.83	Retrosp.	73 (30–93)	d1–5 (15 %) d1–7 (53 %) 5-2-2 (24 %) others (7 %)	66 %, 75 mg/m^2^ 34 %, 100 mg flat	4 (1–37)	8.4	9.6	48 17, 13, 19	130 172	Int: 67^a^ High: 19

*AAR* Austrian Azacitidine Registry, *subanal.* subanalysis, *AZA* azacitidine, *retrosp*. retrospective, *FU* follow-up, *mo* months, *OS* overall survival, *ORR* overall response rate, *CR* complete response, *mCR* marrow complete response, *PR* partial response, *HI* hematologic improvement, *ND* not determined, *BM* bone marrow, *WHO* World Health Organization, *Int.* intermediate, *ND* not done, *MPD* myeloproliferative disease

^a^MRC-criteria, MRC cytogenetic risk groups, Medical research Council cytogenetic risk groups

^b^One hundred eight participating centers from 15 countries (http://clinicaltrials.gov/ct2/show/study/NCT00071799?show_locs=Y#locn)

^c^ISCN criteria, International System for Cytogenetic Nomenclature

^d^Ranges are given, as no separate subanalysis of AML patients was performed. OS ranges thus comprise MDS and AML patients

^e^Merely defined as normal, simple, and complex cytogenetic abnormalities

^f^IPSS cytogenetic risk criteria, IPSS cytogenetic risk groups, International Prognostic Scoring Index cytogenetic risk groups

^g^Baseline characteristics were reported from 52 patients, but only 38 were evaluated for response (14 patients were excluded from survival analyses since they did not reach the minimal 8-week observation period)

^h^Patient recruitment area 2.0 million (as defined by http://www.nzz.ch/aktuell/schweiz/wir-erleben-einen-voelligunethischen-wettbewerb-1.16961164)

^i^HOVON classification

## Patients, design, and methods

Between February 2009 and October 2013, 302 AML patients from 14 specialized centers for hematology and medical oncology in Austria were included. Data cleaning/survival analysis cutoff date was 21 January 2014. The sole inclusion criteria were the diagnosis of WHO-AML and treatment with at least one dose of azacitidine. No formal exclusion criteria existed, as the aim was to include all AML patients treated with azacitidine, irrespective of age, comorbidities, and/or number of previous lines of treatment. Informed consent to allow the collection of personal data was obtained for all retrospectively documented patients who were alive, as well as for all prospectively included patients.

Registry design, data collection and monitoring, as well as assessment of efficacy, safety and endpoints within the Austrian Azacitidine Registry were performed as previously described [8, 15].

Overall response was defined as complete response (CR), marrow complete response (mCR), partial response (PR) (as defined by commonly used AML response criteria [16]), and hematologic improvement (HI) (as defined by the IWG-MDS-2006 response criteria [17]). Marrow stable disease (mSD) was defined as failure to achieve at least PR, but no evidence of progressive disease (PD) for >8 weeks. PD was defined as any of the following: (i) ≥50 % decrement from maximum remission/response in granulocytes or platelets, (ii) reduction in hemoglobin by 2 g/dl, (iii) transfusion dependence [17].

OS was assessed using the Kaplan–Meier method. Univariate analyses were performed with log-rank tests. Cox regression stratified on the various factors was used for analyses of risk factors for OS. Baseline characteristics were compared by nonparametric tests (Chi-squared test for qualitative variables, Wilcoxon test for quantitative variables). For multivariate analysis, logistic regression according to the Wald method with forward stepwise selection (entry level 0.05; level for keeping the variable 0.051) was used. Univariate analyses were performed and confirmed by two independent statisticians [H.A., O.E.]. The confirmed results were the basis for multivariate analysis. All variables with *p* < 0.05 in univariate analyses were included in multivariate analysis, except for those cases, where parsimony would have been disrupted due to redundancy in the variables. Analyses were performed with SPSS. No adjustments were made for multiple testing.

## Results

### Patient characteristics

Patient baseline characteristics at azacitidine treatment start can be taken from Table 2. Median age was 73 (range 30–93); 43 % of patients were older than 75, 21 % were older than 80, and 8 % were older than 85 years, respectively; 172 patients (57 %) had >30 % BM blasts; 19 % had an unfavorable karyotype, and 67 % had an intermediate karyotype according to MRC criteria [18]. In the absence of consensus for cytogenetic classification of AML in the elderly [4, 19], we additionally assessed the IPSS cytogenetic risk categories [20] (Table 2).

**Table 2:** Baseline characteristics at azacitidine treatment start

Median age, years (range)	73 (30–93)
Gender, male, *n* (%)	175 (57.9)
WHO diagnosis, *n* (%)^a, b^	
t-AML	24 (7.9)
AML-RCA^c^/gene mutations^d^	61 (20.2)
AML-RCA	13 (4.3)
AML with gene mutations	52 (17.2)
AML-MRF	203 (67.2)
AML-MRC	75 (22.2)
AML with antecedent hematologic disease	89 (29.5)
Antecedent MDS	60 (19.9)
Antecedent CMML	11 (3.6)
Antecedent CMPD	18 (6.0)
AML with myelodysplasia (MLD)	173 (57.3)
AML-NOS	61 (20.2)
Peripheral blood blasts, *n* (%)	
No data	14 (4.6)
0 %	101 (33.4)
>0 %	187 (61.9)
Median (range), %	3.5 (0–97)
Bone marrow blasts, *n* (%)	
<20 %^e^	51 (16.9)
20–30 %	79 (26.2)
>30 % (off-label use)	172 (57.0)
Median (range), %	32 (0–98)
WBC count, *n* (%)	
<10 G/l	150 (49.7)
≥10 G/l	61 (20.2)
≥15 G/l	40 (13.2)
≥20 G/l	29 (9.6)
≥30 G/l	15 (5.0)
≥50 G/l	7 (2.3)
Transfusion dependence, *n* (%)	
Any type of TD	183 (60.6)
RBC-TD	175 (57.9)
PLT-TD	113 (37.4)
RBC-TD + PLT-TD	105 (34.8)
IPSS cytogenetic risk, *n* (%)	
Not evaluable	33 (10.9)
Good	161 (53.3)
Intermediate	55 (18.2)
Poor	53 (17.5)
MRC cytogenetic risk, *n* (%)	
Not evaluable	33 (10.9)
Good	11 (3.6)
Intermediate	201 (66.6)
High	57 (18.9)
Specific chromosomal aberrations, *n* (%)	
Not evaluable/not evaluated	33 (10.9)
Normal karyotype	149 (49.3)
Specific aberrations^b^	120 (39.7)
Complex karyotype	31 (10.3)
Monosomal karyotype	32 (10.6)
MK only	6 (2.0)
MK and complex	26 (8.6)
−5q	33 (10.9)
−7	25 (8.3)
−7q	17 (5.6)
+8	26 (8.6)
−20q	5 (1.7)
−Y	8 (2.6)
Others	79 (26.2)
Molecular diagnostics^b^, *n* (%)	
Not done/no data	148 (49.0)
Normal	89 (29.5)
FLT3	43 (14.2)
NPM1	36 (11.9)
nv(16)	4 (1.3)
Others	91 (30.1)
Comorbidities^b^, *n* (%)	
Cardiac^e^	124 (41.5)
Renal insufficiency	54 (17.9)
Diabetes mellitus	54 (17.9)
Solid tumor	41 (13.6)
Liver disease	35 (11.6)
Pulmonary	33 (10.9)
Hematologic neoplasm^f^	31 (10.3)
Thromboembolic episodes	28 (9.3)
Infection	28 (9.3)
Obesity (BMI >35 kg/m^2^)	25 (8.3)
Cerebrovascular disease	25 (8.3)
Psychiatric disturbance (requiring consult/treatment)	20 (6.6)
Rheumatologic	11 (3.6)
Peptic ulcer (requiring treatment)	8 (2.6)
Inflammatory bowel disease	3 (1.0)
Number of comorbidities, *n* (%)	
0	62 (20.5)
1	90 (29.8)
2	73 (24.2)
3	43 (14.2)
>3	34 (11.3)
ECOG ≥2, *n* (%)	73 (24.2)
HCT-CI, *n* (%)	
Low risk	93 (30.8)
Int. risk	117 (38.7)
High risk	92 (30.5)
Treatment prior to AZA^g^, *n* (%)	
None	115 (38.1)
Growth factors and/or iron chelators	24 (7.9)
Prior disease modifying treatment	163 (54.0)
Treatment prior to AZA^b^, *n* (%)	
None	115 (38.1)
Erythropoietin stimulating agents	23 (7.6)
G-CSF	34 (11.3)
Thrombopoietin-stimulating agents	2 (0.7)
Iron chelation therapy	12 (4.0)
Thalidomide	5 (1.7)
Lenalidomide	11 (3.6)
ATG, CyA	4 (1.3)
Low-dose cytarabine	13 (4.3)
Intensive chemotherapy for MDS/AML	125 (41.4)
Chemotherapy for other neoplasm	22 (7.3)
Hydroxyurea	26 (8.6)
Others	14 (4.6)
Reason for treatment, *n* (%)	
1st line treatment^h^	139 (46.0)
Bridging to allo-SCT	10 (3.3)
Maintenance after CR to CTX	13 (4.3)
No CR to conventional chemotherapy/allo-SCT	98 (32.5)
No CR to other disease modifying treatment	42 (13.9)

*t-AML* treatment-related AML, *AML-RCA* AML with recurrent cytogenetic abnormalities, *AML-MRF* AML with MDS-related features, *CMPD*, chronic myeloproliferative disease, *AML-NOS* AML not otherwise specified, *MP* myeloproliferative, *COPD* chronic obstructive pulmonary disease, *NHL* non-Hodgkin’s lymphoma, *ECOG* Eastern Cooperative Oncology Group

^a^If a patient fulfilled criteria for more than one WHO category, weighting was performed as follows: t-AML > AML-RCA > AML-MRF

^b^Amounts to >100 % due to multiple choice nature

^c^Includes the following structural abnormalities: inversion 16, t(8;21)**,** t(15;17)**,** t(9;11), t(6;9)**,** t(1;22)

^d^Includes mutations in FLT3, NPM1, and/or CEBPα

^e^Includes arrhythmia (atrial fibrillation or flutter, sick sinus syndrome, or ventricular arrhythmias), coronary artery disease, coronary heart disease, myocardial infarction or ejection fraction ≤50 %, and/or valvular heart disease (except mitral valve prolapse)

^f^Includes monoclonal gammopathy of unknown significance, multiple myeloma, low-grade NHL, high-grade NHL, M. Hodgkin, Burkitt’s lymphoma, chronic myeloid leukemia, hypereosinophilic syndrome, chronic myeloproliferative diseases, and others

^g^BM blast count was <20 %, in those patients with established AML who were refractory to -or had no CR after-conventional chemotherapy or allogeneic stem cell transplantation

^h^Defined as patients without prior disease modifying treatment (i.e., growth factors and iron chelation were allowed)

### Treatment modalities

Azacitidine was administered as first-line treatment in 46 % of patients; 46 % of patients received azacitidine after insufficient response to, or early relapse after, conventional chemotherapy/allo-SCT (32 %), or other disease modifying treatment (14 %); the remaining patients received azacitidine either as bridging to allogeneic stem cell transplantation (allo-SCT) (3 %) or as maintenance treatment after CR to chemotherapy (4 %). Azacitidine was not always first-line therapy, but also second-, third-, fourth-, fifth-, or last-line therapy for a relevant proportion of our patients: 28 % had received more than one line of conventional chemotherapy prior to azacitidine (Table 2).

A median number of 4 (range 1–37) azacitidine courses were given. Most patients (85 %) received the drug subcutaneously (average dose/cycle, 846 mg), 10 % intravenously (average dose/cycle, 815 mg), and in 5 % both applications forms were used (average dose/cycle, 831 mg); 77 % of patients predominantly received 7 days of azacitidine (53 % European Medicines Agency (EMA)-approved d1-7 (median dose/cycle, 924 mg), 24 % 5-2-2 (median dose/cycle, 910 mg)) (Supplemental Table 1). EMA-approved azacitidine target dose (75 mg/m^2^ × 7 ± 10 %) was reached in 33 % of applied cycles; 349/2013 (17 %) of all cycles were administered as “flat” dosage (i.e., 100 mg azacitidine/cycle-day; median dose/cycle, 700 mg). Hospitalization for the sole reason of azacitidine application occurred in 47 % of cycles at the discretion of the treating physician, mainly due to concerns with frailty and potential toxicity, and due to logistic reasons for patients living far away from the hospital. Azacitidine treatment dose was modified during treatment in 18 % of patients: dose reductions occurred due to an adverse event (15 %) or due to patient’s/physicians wish (2 %); dose escalation was performed in 1 % of patients. Dose reductions were performed in 32 % of responding patients (prior to best response (13 %), at best response (3 %), or after best response (15 %)).

### Concomitant treatment and best supportive care measures

Erythropoietin-stimulating agents (ESA) (2.4 %), iron chelation treatment (ICT) (2.3 %), and G-CSF (18.4 %) were given in parallel to azacitidine when deemed necessary by the treating physician.

### Response

Overall response, defined as CR, mCR, PR, and HI, was documented in 48 % of the total intention-to-treat (ITT) cohort and in 72 % of patients evaluable according to MDS-IWG-2006 response criteria [17] (i.e., had received >2 cycles of azacitidine); HI was documented in 40 % (ITT) and 72 % (evaluable according to IWG, i.e., had received >2 cycles of azacitidine), respectively; CR/mCR was achieved in 17 % of the ITT cohort and in 28 % of patients in whom a BM aspirate/biopsy was performed (Supplemental Table 2).

The median number of cycles received by responding patients was 8.5 (range 1–37), and 2 (range 1–28) in non-responders (included patients with SD). Of note, the distribution of applied schedules as well as the median and mean azacitidine dosages/cycle did not differ between responders and non-responders.

### Time to response and response deepening

Median time to first response was 3.0 months. First response occurred after 3, 4, and 5 cycles in 58, 79, and 88 % of responding patients, respectively, but could be observed as late as cycle 16 in patients with stable disease who were kept on therapy. First response was best response in 99/144 patients (69 %). Median response duration was 3.4 (range 0.3–33.0) months. Further deepening of response after first response (i.e., achievement of BM blast reduction in terms of mCR/CR/PR after HI) was seen in 45/144 (31 %) of responders. Best response was reached by cycle 9 in 94 %, but could be observed as late as cycle 21. Median time from first to best response was 3.5 (range 0.8–21.5) months.

### Toxicity and adverse events

A total of 1.031 adverse events (AE) were documented in 2.013 azacitidine cycles. Overall, 24 % of all AE and 20 % of grade 3–4 (G3-4) AE were attributed to azacitidine (Supplemental Table 3). Of all AE, 22 % resulted in hospitalization and 9 % resulted in death. Most AE had no consequence for azacitidine treatment (66 %). Dose reduction (5 %), treatment pause (11 %), prolongation of azacitidine cycle duration >28 days (7 %), and/or termination of azacitidine treatment (11 %) were not commonly necessary.

G3-4 hematologic toxicity occurred in 48 %: G3-4 neutropenia, thrombocytopenia, and anemia were documented in 35, 30, and 28 % of patients, respectively; clinically relevant bleeding events were noted in 12 % of patients (Table 3). Non-hematologic toxicity was usually mild, the most common AE being fatigue (39 %), gastrointestinal (38 %), unspecified pain (30 %), and injection site reactions (22 %). Infectious complications of any grade were documented in 63 %, febrile neutropenia in 19 % (Table 3). G3-4 infectious events occurred in 33 % and were dominated by pulmonary infections, sepsis, and fever of unknown origin. Hospital admission was required in 173/423 (41 %) and transfer to an intensive care unit was necessary in 3 % of total infectious events. Treatment with G-CSF, antibiotics, antifungals, and/or virostatics occurred in 18, 86, 22, and 14 %, respectively.

**Table 3:** Specific adverse events^a^

Variable	Grade	No. of patients, (%)	No. of total events
Hematologic toxicity^b^	G3-4	145 (48.0)	330
Thrombopenia	G3-4	91 (30.1)	195
Neutropenia	G3-4	105 (34.8)	223
Anemia	G3-4	84 (27.8)	177
Bleeding events	–	35 (11.6)	60
Febrile neutropenia	–	56 (18.5)	95
Infectious complications	G1-2	91 (30.1)	311
	G3-4	100 (33.1)	112
Non-hematologic toxicity			
Liver	G1-2	2 (0.7)	3
	G3-4	2 (0.7)	2
Kidney	G1-2	12 (4.0)	12
	G3-4	3 (1.0)	3
Heart^c^	G1-2	8 (2.6)	14
	G3-4	29 (9.6)	34
Blood pressure	G1-2	3 (1.0)	3
	G3-4	5 (1.7)	5
Metabolic	G1-2	4 (1.3)	4
	G3-4	0 (0.0)	0
Thromboembolic	G1-2	11 (3.6)	12
	G3-4	2 (0.7)	2
Neurologic	G1-2	19 (6.3)	26
	G3-4	2 (0.7)	2
Nausea	G1-2	33 (10.9)	46
	G3-4	1 (0.3)	1
Vomiting	G1-2	9 (3.0)	12
	G3-4	0 (0.0)	0
Constipation	G1-2	21 (7.0)	28
	G3-4	1 (0.3)	1
Diarrhea	G1-2	26 (8.6)	35
	G3-4	0 (0.0)	0
Gastrointestinal, others	G1-2	24 (7.9)	31
	G3-4	0 (0.0)	0
Injection site reaction	G1-2	64 (21.2)	123
	G3-4	2 (0.7)	2
Fatigue	Relieved by rest	46 (15.2)	89
	Not relieved by rest	36 (11.9)	63
	Limiting self care	37 (12.3)	42
Pain	Mild	43 (14.2)	82
	Moderate	39 (12.9)	51
	Severe	9 (3.0)	9
Surgery	Elective	19 (6.3)	23
	Emergency	10 (3.3)	11
Fall	Total	26 (8.6)	29
	With fracture	10 (3.3)	11
	With hemorrhage	11 (3.6)	11
Novel solid tumor	Yes	3 (1.0)	3

^a^Assessed according to NCI Toxicity Criteria (http://ctep.cancer.gov/protocolDevelopment/electronic_applications/ctc.htm) and Common Terminology Criteria for AE (CTCAEv.4) (http://evs.nci.nih.gov/ftp1/CTCAE/About.html)

^b^Grade 3–4 cytopenias reported, are those that were documented as adverse events, and thus felt to be a worsening of pre-existing cytopenia by the respective treating physicians

^c^Reported cardiac AE were left ventricular output failure (*n* = 23), arrhythmia (*n* = 7), hypertension (*n* = 5), myocardial infarction (*n* = 3), angina pectoris (*n* = 1)

A total of 47 non-hematologic G3-4 events occurred in 33 patients (11 %): 39 (83 %) of these grade 3–4 events occurred in the cardiac system: left ventricular output failure (*n* = 23), arrhythmia (*n* = 7), hypertension (*n* = 5), myocardial infarction (*n* = 3), angina pectoris (*n* = 1). In 20/33 (61 %) patients experiencing cardiac G3-4 events, pre-existing coronary artery disease, reduced cardiac function, arrhythmias, and/or valvular heart disease were documented prior to azacitidine treatment and worsening was not thought to be azacitidine-related.

### Overall survival and potential prognostic parameters

Median OS was 9.6 (95 % CI 8.53–10.7) months as from initiation of treatment with azacitidine in the entire cohort. Median progression-free survival in responding patients was 9.1 (0.9–39.9) months. Progression defining events were death due to any reason, disease progression, disease relapse after response, and/or new cytogenetic aberration/clonal evolution. Median OS was 16.1 months for responders (defined as CR/mCR/PR/HI) and 3.7 months for non-responders. Median time from first diagnosis to treatment start with azacitidine for untreated (*n* = 139) versus pre-treated with disease-modifying treatment (*n* = 163) patients was 0.6 and 7.1 months, respectively. Median time from azacitidine treatment stop to death was 1.9 months in the entire cohort. Reasons for cessation of azacitidine in patients receiving ≤2 cycles of azacitidine (*n* = 101, 33.4 %) were death (*n* = 48), disease progression (*n* = 20), patient’s wish (*n* = 9), toxicity or recurrent infectious complications (*n* = 5), allo-SCT (*n* = 2), and others (*n* = 13); 41/101 receiving ≤2 cycles died within 1 month of treatment termination, and a further 35 died within 6 months.

**Fig. 1:**
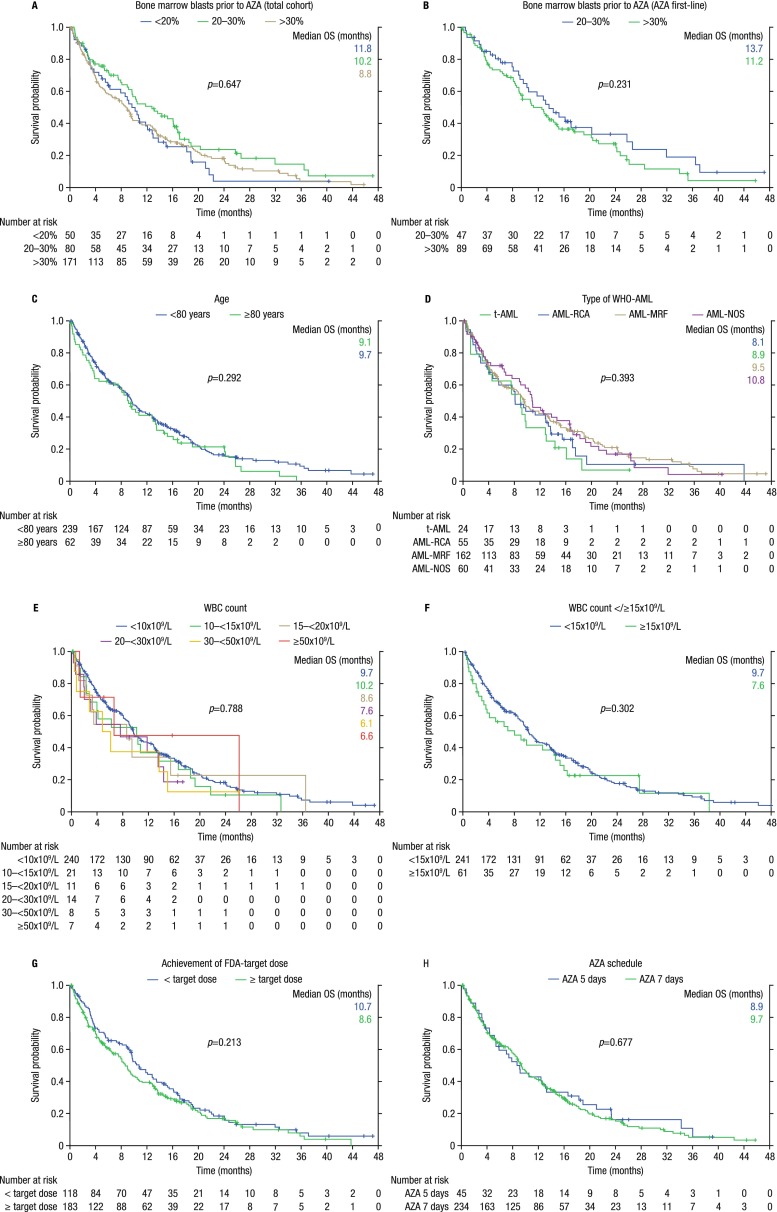
Kaplan–Meier curves of baseline factors that did not affect overall survival (OS). **a** Effect of bone marrow blast count on OS (total cohort). **b** Effect of bone marrow blast count on OS (AZA first line). **c** Effect of age on OS. **d** Effect of WHO-AML type on OS. **e** Effect of white blood cell (WBC) count on OS. **f** Effect of WBC </≥15 G/l on OS. **g** Effect of achievement of EMA/FDA-target dose on OS. **h** Effect of azacitidine (AZA) schedule: 5 vs. 7 days of azacitidine per cycle on OS

In line with our previous results [8], the following baseline factors did not significantly affect overall survival: gender, age </≥75, age </≥80, WHO-AML-type, WBC count </≥10 G/l, WBC count </≥15 G/l, WBC count </≥30 G/l, neutrophil count <1,000/μl, lymphocyte count <2,000/μl, RBC-TD, BM blast count ≤30/>30 % (irrespective of whether the whole cohort or only patients treated with azacitidine first line were analyzed), serum erythropoietin level, as well as prior treatment with ESA, G-CSF, iron chelators, low-dose Ara-C, or hydroxyurea (Supplemental Table 4 and Fig. 1a–h). When looking at responding patients only, WHO-AML type, WBC count, and treatment according to EMA label had no significant effect on survival (Supplemental Table 4).

Time-dependent factors that did not affect overall survival include the following: EMA target dose (*p* = 0.213), treatment schedule, dose/cycle, and platelet-doubling after one cycle (Supplemental Table 4). The following toxicity and adverse events-related factors had no effect on overall survival: bleeding events, febrile neutropenia, surgery, non-hematologic toxicity, falls, and pain due to any reason (Supplemental Tables 1 and 4).

**Fig. 2:**
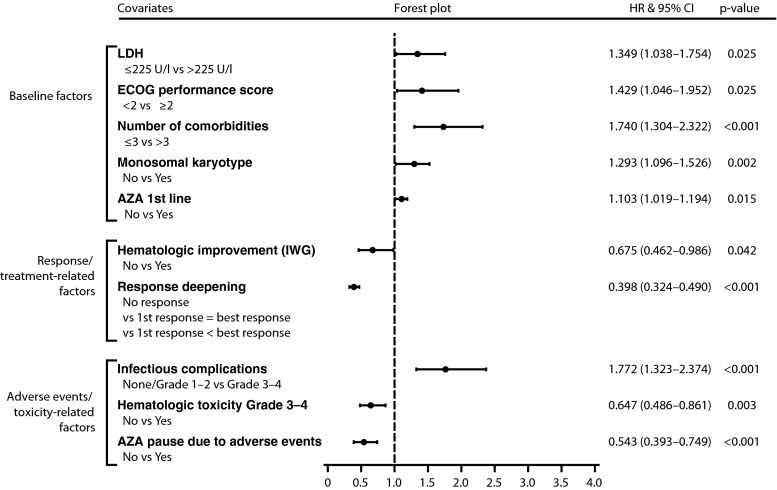
Forrest plot of factors significantly influencing overall survival of azacitidine-treated AML patients (*n* = 302) in multivariate analysis

**Fig. 3:**
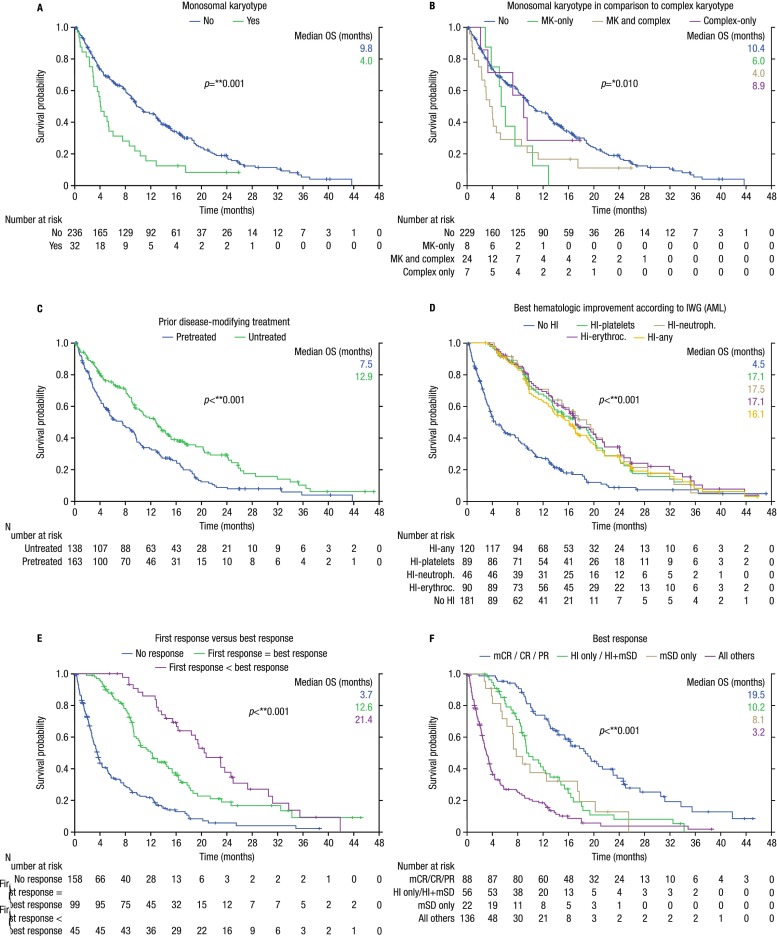
Kaplan–Meier curves of baseline factors that significantly affected overall survival (OS) in multivariate analysis. **a** Effect of monosomal karyotype (MK) on OS. **b** Effect of MK in comparison to complex karyotype on OS. **c** Effect of prior disease-modifying treatment (i.e., azacitidine first-line no vs. yes) on OS. **d** Effect of hematologic improvement (HI) on OS. **e** Effect of response deepening (i.e., achievement of BM blast reduction in terms of mCR/CR/PR after HI) on OS. **f** Overall survival by best response

In multivariate analysis, the following baseline factors remained independent adverse predictors for OS: LDH >225 U/l, ECOG ≥2, number of comorbidities (as predefined by the HCT-CI [21]) >3, and monosomal karyotype. The following treatment-related factors that remained independent predictors for OS in multivariate analysis were as follows: AZA first-line treatment, hematologic improvement, and further deepening of response after first response; best marrow response (*p* = 0.642) did not meet the 0.05 significance level for inclusion in the multivariate analysis; azacitidine pause due to adverse events was associated with longer OS in multivariate analysis; dose reduction due to adverse events (*p* = 0.051) and fatigue limiting self-care (*p* = 0.053) were borderline significant in multivariate analysis (Figs. 2 and 3, and Supplemental Table 5).

## Discussion

This is the largest so far published number of azacitidine-treated WHO-AML patients, with the highest per capita coverage of AML patients in a nationwide registry, suggesting limited selection (Table 1, Supplemental Table 7). Data on the efficacy of azacitidine in AML with >30 % BM blasts are limited, and the drug can only be used off-label in these patients (Table 1 [4–14]). We report on 302 WHO-AML patients treated with azacitidine. This cohort included 172 patients with >30 % BM blasts (Tables 1 and 4 [6–8, 12, 14]). The number of AML diagnoses per year, as well as the number of AML patients included in the respective year for Austria in general and Salzburg in particular, is presented in Supplemental Table 7 (data obtained from Statistics Austria (personal communication 23 May 2014), and Tumor Registry Salzburg (personal communication 26 May 2014)).

**Table 4:** Comparison of prognostic factors for OS in multivariate analysis (MVA) of all full publications on azacitidine treated AML patients

Variable	Italy	Holland	Lausanne	France	Austria	Present study
*n* AML patients	82	55	38^a^	149	155	302
*n* AML patients with >30 % BM blasts	49	17	29	87	98	172
Prognostic factors for OS						
Age	No MVA for OS performed	ND	No	No	No	No
Gender		ND	No	No	No	No
Cytogenetic risk group		Yes (**p = 0.001)^b, c^	No^d^	Yes (**p < 0.001)^b^	No^b^	No^b^
Monosomal karyotype		ND	ND	ND	ND	Yes (**p = 0.002)
WBC </≥15 g/l		Yes (**p = 0.003)^e^	ND	Yes (**p = 0.001)	No	No
LDH </≥225 IU/l		No	ND	ND	No	Yes (*p = 0.025)
ECOG performance score </≥2		Yes (**p = 0.006)^f^	ND	Yes (**p = 0.006)	Yes (*p = 0.040)	Yes (*p = 0.025)
Number of comorbidities </≥4		ND	ND	ND	No (*p* = 0.086)	Yes (**p < 0.001)
AZA first line		ND	ND	ND	ND	Yes (*p = 0.015)
AML type^g^		No	No^h^	No	No	No
BM blasts </≥30 %		No	No	No	No	No
PB blasts </≥0 %		No	No^i^	ND	Yes (*p = 0.040)	No
Transfusion dependence		No	Yes (**p = 0.009)^j^	ND	No	No
RBC-TI		ND		ND	Yes (**p < 0.001)	Yes (*p = 0.042)
PLT-TI		ND	No	ND		
Hematologic improvement		ND	ND	ND		
AZA schedule (5 vs. 7 days)		ND	ND	No	No	No
AZA dose (</=75 mg/m^2^/day)		ND	ND	No	No	No

^a^Baseline characteristics were reported from 52 patients, but only 38 were evaluated for response

^b^Defined according to MRC classification

^c^Only the poor risk-group predicted OS, the good vs. intermediate risk group did not (*p* = 0.91)

^d^Defined according to the HOVON classification

^e^Cutoff, </>10 G/l; however, this variable was not significant in univariate analysis (*p* = 0.11)

^f^According to WHO performance score

^g^Primary AML, post-MDS, post-MPN, refractory/relapsed disease or therapy-related AML

^h^Only therapy-related AML (*n* = 10 patients) had an impact when looked at separately (**p < 0.001), whereas AML type did not (*p* = 0.086)

^i^PB blast cut off of ≥20 % used

^j^Not reported on separately, i.e., patients that were transfusion independent at baseline, or who achieved RBC-TI during treatment were grouped together

In order to exclude a potential time-dependent bias regarding both the choice of AML patients for treatment with azacitidine, as well as inclusion in the Austrian Azacitidine Registry, we compared all relevant baseline characteristics, treatment modalities, as well as response rates, and the occurrence of adverse events of our previously published cohort (*n* = 155; data cutoff 21 January 2012) [8] with the current cohort (*n* = 302, data cut off 21 January 2014). No significant difference could be found for any of these characteristics (Supplemental Table 6). With this nearly twice as large cohort, we confirm and validate the safety and efficacy of azacitidine in WHO-AML patients treated in a real-life setting. The observed median OS of 9.6 months and the high overall response rates (48 % ITT) seem remarkable, particularly since (i) the registry included many elderly (43 % ≥75 years), comorbid (79 %), and/or pretreated patients (62 %); (ii) 33 % of patients received ≤2 cycles of azacitidine (reasons therefore listed in the “Results” section); (iii) 82 % of our registry population would have been excluded from the AZA-001 registration trial due to an estimated life expectancy <3 months, ECOG >2, t-AML, prior treatment, or planned allo-SCT [3, 4]. If BM blast count >30 % is also taken into account, 93 % would have been excluded.

Furthermore, virtually all of our prior results [8] regarding the statistical value of various factors on OS are confirmed by the present analysis. Slight discrepancies are mentioned below and in Table 4. In addition to the parameters looked at previously, we analyzed the putative prognostic effect of platelet-doubling after cycle 1, monosomal karyotype, and disease stabilization, as these factors have emerged to be of interest only recently [12, 13, 22].

This is the first report to analyze the effect of platelet-doubling after cycle 1 on OS in AML patients treated with azacitidine. Statistical significance was not observed (Supplemental Table 4).

We confirm the recently introduced monosomal karyotype (MK) [23] to be the strongest adverse cytogenetic predictor for OS (even outperforming the categories “complex karyotype” and “MDS-related cytogenetic abnormalities” [22–24]) (Figs. 2 and 3a–b, Supplemental Table 5). We extend these findings to the new WHO classification and show that azacitidine cannot overcome the adverse outcome conferred by the presence of MK. Outcome in patients with MK is not only dismal with azacitidine but also with induction therapy and/or allogeneic stem cell transplantation [22]. Therefore, clinical trials are urgently needed for this patient subgroup.

We confirm our previous results [8] that a certain amount of aplasia induction seems necessary before response occurs, and that further deepening of response after first response (i.e., achievement of BM blast reduction in terms of mCR/CR/PR after HI) translates into significantly longer OS (21.4 months), compared with patients for whom first response was best response (12.6 months) (Figs. 2 and 3e, Supplemental Table 5). This underlines the importance of continuing azacitidine treatment if or when HI occurs, even in the absence of marrow response. Patients experiencing HI had significantly longer OS than those who did not (16.1 vs. 4.5 months) (Fig. 3d and Supplemental Table 5). This is the first report to separately analyze HI and marrow response in multivariate analysis. The former remained an independent prognostic factor for OS in multivariate analysis, whereas the latter did not (Fig. 2 and Supplemental Table 5). This seems of clinical relevance, as hematologic improvement is not generally considered sufficient response in AML patients. Very recently, the French group reported no survival benefit for patients achieving HI in addition to stable disease [12]. However, the authors state that (i) only those patients not achieving mCR/CR/PR were analyzed for HI; (ii) HI was only assessed when patients were evaluable for these parameters; (iii) it is not stated how many patients were not evaluable for HI or how SD was defined (i.e., marrow SD or hematologic SD). In our cohort, OS was significantly better for patients achieving mCR/CR/PR with HI (20.5 months), followed by mSD with HI (18.9 months), mCR/CR/PR without HI (15.0 months), HI without mSD (9.7 months), mSD without HI (8.1 months), and not unexpectedly, OS was worst for patients with progressive disease or who received less than 3 cycles of azacitidine (3.2 months) (Supplemental Table 5). We thus confirm and extend the observations of the French group [12] as follows: It seems that achievement of any form of disease stabilization, be it mSD or HI alone, and especially the combination thereof (without the requirement of concomitant BM blast reduction, but with the minimal requirement of mSD), is sufficient to confer a clinically relevant OS benefit. In our opinion and clinical experience, we thus consider disease stabilization to be sufficient “response” to continue treatment with azacitidine, although we are aware of the fact that this is currently not regarded as a standard form of response assessment in AML (Fig. 3f). In our experience, disease stabilization is lost rapidly once treatment with azacitidine is stopped (median time to death, 1.9 months). Thus bearing the lack of therapeutic alternatives in mind, we continue azacitidine treatment until overt clinical disease progression.

In order to facilitate the comparison of our previous and current results with data obtained from much smaller cohorts, we provide an in-depth comparison of all published multivariate analyses on putative prognostic factors for OS of WHO-AML patients treated with azacitidine (including results from the present study) (Table 4). Only one baseline factor remained a significant adverse predictor for OS in all multivariate analysis (4/4) in which baseline factors were analyzed, namely ECOG/WHO performance score (Table 4). Thus, the ability of patients to function in everyday life seems to be the most important predictor of OS in elderly AML patients treated with azacitidine. In line with this, the absolute number of comorbidities, which was borderline significant in our previously published smaller cohort of AML patients (*n* = 155) [8], was confirmed to be an independent adverse predictor of OS in our larger present cohort (*n* = 302) (Fig. 2, Table 4, and Supplemental Table 5).

Although not unexpected, we show for the first time in multivariate analysis—thus confirming our clinical experience and notion—that prior disease-modifying treatment is an adverse predictor of OS for AML patients treated with azacitidine. In multivariate analysis, patients receiving azacitidine first line had significantly longer overall survival than pretreated patients (12.9 vs. 7.5 months) (Figs. 2 and 3c, Supplemental Table 5).

Age, gender, AML type, and transfusion dependence prior to azacitidine had no significant effect on OS in all reports in which these factors were analyzed (Table 4, Supplemental Table 4, and Fig. 1c, d). As azacitidine is still not approved for the treatment of AML patients with >30 % BM blasts, we were particularly interested to see that the percentage of BM blasts had no significant effect on OS in this off-label treated patient subgroup (*n* = 172), which is in line with observations by others with smaller patient numbers (Fig. 1a, b, Table 4, and Supplemental Table 4).

The present report confirms that WHO-AML type does not seem to adversely predict OS. We conclude that AML patients with MDS-related features, or therapy-related AML, should not be precluded from treatment with azacitidine. The latter seems clinically relevant, especially in light of the fact that these AML subgroups are generally considered to have worse prognosis and to be less responsive to conventional chemotherapy.

The prognostic relevance of elevated WBC ≥15 G/l currently remains unclear and conflicting results exist (Table 4). Several statistical issues remain open in the two publications describing a significant adverse effect of WBC ≥15 G/l on OS in multivariate analysis: (i) in the Dutch cohort, this variable was not significant in univariate analysis (*p* = 0.11), but was nevertheless included in multivariate analysis. Furthermore, 95 % CI were not given for multivariate results [14]; (ii) the univariate *p* values used for entry of the variables into the multivariate model (*p* < 0.15 [14] and *p* < 0.85 [25], respectively) were less stringent than in the present publication (*p* < 0.05), which could not find an adverse effect of elevated WBC, irrespective of which cutoff value was used (</≥10, </≥15, or </≥30 G/l (Supplemental Table 4 and Fig. 1e, f)).

Neither azacitidine schedule (5 vs. 7 days) nor dosage (<vs. = 75 mg/m^2^/day) had a significant effect on OS in 3/3 reports in which these factors were analyzed (Table 4, Supplemental Table 4, and Fig. 1g, h). Thus, alternative schedules and dosages seem safe and without loss of efficacy, and receiving the drug regularly and continuously until overt clinical progression occurs is likely more relevant than the absolute dosage per day or number of days per cycle.

In conclusion, we confirm that azacitidine is safe and effective in elderly, comorbid AML patients treated in an everyday life setting, irrespective of BM blast count.

## Electronic supplementary material

Below is the link to the electronic supplementary material.

Supplemental Table 1 (DOCX 23 kb)

Supplemental Table 2 (DOCX 24 kb)

Supplemental Table 3 (DOCX 22 kb)

Supplemental Table 4 (DOCX 30 kb)

Supplemental Table 5 (DOCX 36 kb)

Supplemental Table 6 (DOCX 37 kb)

Supplemental Table 7 (DOCX 24 kb)
