# Supplementary material for: Azacitidine in 302 patients with WHO-defined acute myeloid leukemia: results from the Austrian Azacitidine Registry of the AGMT-Study Group
Source: Ann Hematol. 2014 Jun 21;93(11):1825–38. doi: 10.1007/s00277-014-2126-9 (PMC4176957; doi:10.1007/s00277-014-2126-9)
Supplement: Supplementary file 1 — (DOCX 23 kb) [file 277_2014_2126_MOESM1_ESM.docx]

**Supplemental Table 1. Azacitidine treatment schedule**

| **Variable** | **n pts.^1^, (%)** | **Mean dose,**  **mg** | **Median dose,**  **mg** | **Dose range,**  **mg** |
| --- | --- | --- | --- | --- |
| **AZA schedule all patients (n=302)**  AZA 1–5  AZA 1–7  AZA 5-2-2  AZA others | 46 (15.2)  161 (53.3)  73 (24.1)  22 (7.3) | 697  902  892  625 | 688  924  910  600 | 350-1035  437-1190  455-1181  300-1120 |
| **AZA schedule responders (n=144)**  AZA 1–5  AZA 1–7  AZA 5-2-2  AZA others | 21 (7.0)  72 (23.8)  41 (13.6)  10 (3.3) | 727  885  869  591 | 715  921  903  586 | 375-1000  437-1100  455-1181  300-1120 |
| **AZA schedule non-responders (n=158)**  AZA 1–5  AZA 1–7  AZA 5-2-2  AZA others | 25 (8.3)  89 (29.5)  32 (10.6)  12 (4.0) | 672  915  920  653 | 650  928  931  650 | 350-1035  469-1190  518-1151  300-900 |

AZA indicates azacitidine;

^1^Refers to patients who predominantly had this type of azacitidine schedule
