# Supplementary material for: Azacitidine in 302 patients with WHO-defined acute myeloid leukemia: results from the Austrian Azacitidine Registry of the AGMT-Study Group
Source: Ann Hematol. 2014 Jun 21;93(11):1825–38. doi: 10.1007/s00277-014-2126-9 (PMC4176957; doi:10.1007/s00277-014-2126-9)
Supplement: Supplementary file 2 — (DOCX 24 kb) [file 277_2014_2126_MOESM2_ESM.docx]

**Supplemental Table 2. Response to azacitidine**

|  | **n(/n) (% ITT)** | **n(/n) [% IWG**^1^**]** |
| --- | --- | --- |
| **Transfusion independence**  PLT-TI  RBC-TI | 47/113 (41.6)  68/175 (38.9) | 47/76 [61.8]  68/115 [59.1] |
| **Hematologic improvement**  HI-Any  HI-platelets  HI-neutrophils  HI-erythrocytes  No HI | 120/302 (39.7)  89/302 (29.5)  46/302 (15.2)  90/302 (29.8)  182/302 (60.3) | 120/201 [59.7]  89/201 [44.3]  46/201 [15.2]  90/201 [29.8]  81/201 [40.3] |
| **Best marrow response**^2^  CR  mCR  PR  mSD  primary PD | 38/302 (12.6)  12/302 (4.0)  38/302 (12.6)  34/302 (11.3)  13/302 (4.3) | 38 [28.1]  12/135 [8.9]  38/135 [28.1]  34/135 [25.1]  13/135 [9.6] |
| **Overall response**^3^  Yes  No response | 144/302 (47.7)  158/302 (52.3) | 144/201 [71.6]  57/201 [28.4] |
| **Overall response**^3^  AZA 1st line  AZA non 1st line | 81/139 (58.3)  63/163 (38.7) | 81/109 [74.3]  63/92 [68.5] |

ITT indicates intent to treat analysis; IWG, International Working Group Criteria; PLT, platelet; TI, transfusion independence; RBC, red blood cell; HI, hematologic improvement; CR, complete response; mCR, marrow CR; PR, partial response; SD, stable disease; mSD, marrow stable disease; PD, progressive disease; EMA, European Medicines Agency;

^1^Evaluable according to IWG-criteria, i.e. patients that received >2 cycles of azacitidine (n=201);

^2^Concerns patients in whom bone marrow puncture was performed; bone marrow assessment was performed as clinically necessary, and at the discretion of the respective treating physician; a total of 277 bone marrow punctures were performed in 135 patients; of patients in whom no bone marrow puncture was performed, n=59 received only ,1 and n=42 received only 2 cycles of azacitidine; of these, 67 died within 3 months after stop of treatment with azacitidine;

^3^Overall response was defined as CR, mCR, PR and/or HI;
