# Supplementary material for: Azacitidine in 302 patients with WHO-defined acute myeloid leukemia: results from the Austrian Azacitidine Registry of the AGMT-Study Group
Source: Ann Hematol. 2014 Jun 21;93(11):1825–38. doi: 10.1007/s00277-014-2126-9 (PMC4176957; doi:10.1007/s00277-014-2126-9)
Supplement: Supplementary file 3 — (DOCX 22 kb) [file 277_2014_2126_MOESM3_ESM.docx]

**Supplemental Table 3. Overview of adverse events**^1^ **and toxicity**^2^

| **Variable** | **n events, (%)** |
| --- | --- |
| **AE attributable to azacitidine**  No  Yes  Unknown  Total AE | 651 (63.0)  247 (23.9)  133 (12.9)  1031 (100.0) |
| **G3-4 AE attributable to azacitidine**  No  Yes  Unknown  Total G3–4 AE | 252 (60.1)  82 (19.6)  84 (20.1)  418 (100.0) |
| **AE duration**  < 3days  < 1week  1 < 2 weeks  2 < 3weeks  3 < 4weeks  ≥ 4weeks  Total AE | 265 (25.7)  261 (25.3)  195 (18.9)  87 (8.4)  55 (5.3)  167 (16.2)  1031 (100.0) |
| **Consequence of AE**  None  Treatment  Hospitalization  Life threatening/intensive care unit  Death  Total AE | 361 (35.0)  344 (33.3)  231 (22.4)  9 (0.9)  88 (8.5)  1031 (100.0) |
| **Consequence for azacitidine treatment**^3^  None  Dose reduction  Treatment pause  Termination of azacitidine treatment  Prolongation of cycle > 28days  Total | 697 (65.9)  53 (5.0)  116 (11.0)  114 (10.8)  78 (7.4)  1058 (100.0)^3^ |

AE indicates adverse event; G3-4, grade 3-4;

^1^<http://evs.nci.nih.gov/ftp1/CTCAE/About.html>

^2^National Cancer Institution Toxicity Criteria (<http://ctep.cancer.gov/protocolDevelopment/electronic_applications/ctc.htm>)

^3^Amounts to >195 due to multiple choice nature of the answer
