# Supplementary material for: Azacitidine in 302 patients with WHO-defined acute myeloid leukemia: results from the Austrian Azacitidine Registry of the AGMT-Study Group
Source: Ann Hematol. 2014 Jun 21;93(11):1825–38. doi: 10.1007/s00277-014-2126-9 (PMC4176957; doi:10.1007/s00277-014-2126-9)
Supplement: Supplementary file 4 — (DOCX 30 kb) [file 277_2014_2126_MOESM4_ESM.docx]

**Supplemental Table 4. Factors that did not significantly affect overall survival**

| **Factors not affecting OS** | **n** | **Median OS, days** | ***p-*value**^1^ |
| --- | --- | --- | --- |
| **Baseline factors** |  |  |  |
| **Gender**  Female  Male | 126  175 | 311  273 | 0.343 |
| **Age**  < 75 years  ≥ 75 years | 171  130 | 279  311 | 0.667 |
| **Age**  < 80 years  ≥ 80 years | 239  62 | 293  278 | 0.292 |
| **Age**  < 65 years  65-69 years  70-74 years  75-79 years  80-84 years  ≥ 85 years | 58  50  63  68  37  25 | 232  293  298  359  294  273 | 0.635 |
| **WHO-type**  t-AML  AML-RCA  AML-MRF  AML-NOS | 24  55  161  61 | 272  246  289  329 | 0.393 |
| **WBC-count**  ≤ 10 G/l  > 10 G/l | 240  61 | 294  232 | 0.178 |
| **WBC-count**  ≤ 15 G/l  > 15 G/l | 261  40 | 294  232 | 0.302 |
| **WBC-count**  ≤ 30 G/l  > 30 G/l | 286  15 | 293  202 | 0.668 |
| **Neutrophil count <1.000/µl**  No  Yes | 132  148 | 298  267 | 0.748 |
| **Lymphocyte count <2.000/µl**  No  Yes | 140  110 | 281  272 | 0.972 |
| **RBC-TD prior to AZA**  No  Yes | 127  174 | 297  287 | 0.127 |
| **BM blasts (total cohort)**  < 20%  20-30%  >30% | 51  79  171 | 294  389  267 | 0.120 |
| **BM blasts (AZA 1st line)**  20-30%  >30% | 47  89 | 416  342 | 0.231 |
| **Serum erythropoietin level**  <50 IU/l  50-100 IU/l  <500 IU/l | 22  29  6 | 121  278  294 | 0.279 |
| **Prior ESA**  No  Yes | 278  23 | 289  293 | 0.924 |
| **Prior G-CSF**  No  Yes | 267  34 | 289  309 | 0.852 |
| **Prior ICT**  No  Yes | 296  5 | 291  289 | 0.548 |
| **Low-dose Ara-C**  No  Yes | 288  13 | 287  410 | 0.918 |
| **Prior Hydroxyurea**  No  Yes | 275  26 | 294  165 | 0.065 |
| **Baseline factors in responding patients** | | | |
| **WHO-type (responders)**  t-AML  AML-RCA  AML-MRF  AML-NOS | 11  25  75  33 | 298  470  574  382 | 0.175 |
| **WBC-count (responders)**  <10 G/l WBC  >=10 G/l WBC | 118  26 | 490  456 | 0.327 |
| **Treatment on FDA label^3^ (responders)**  Off-label  On-label | 119  25 | 462  520 | 0.281 |
| **Time-dependent factors - Treatment related factors** | | | |
| **FDA target dose^2^**  < target dose  ≥ target dose | 119  182 | 327  262 | 0.213 |
| **Predominant schedule**  d1–5  d1–7/5-2-2 | 45  234 | 272  294 | 0.677 |
| **Predominant schedule (responders)**  d1–5  d1–7/5-2-2 | 21  113 | 575  455 | 0.511 |
| **Predominant dose/cycle (responders)**  < 800 mg  ≥ 800 mg | 51  93 | 462  489 | 0.816 |
| **Time-dependent factors – Response** | | | |
| **Platelet doubling after one cycle**  No  Yes | 220  23 | 330  574 | 0.141 |
| **Time-dependent factors – Toxicity and adverse events** | | | |
| **Bleeding events**  No  Yes | 267  34 | 287  416 | 0.075 |
| **Febrile neutropenia**  No  Yes | 246  55 | 294  275 | 0.672 |
| **Surgery**  None  Elective  Emergency | 275  16  10 | 279  573  332 | 0.311 |
| **Non-hematologic toxicity**  None  Grade 1–2  Grade 3–4 | 218  44  39 | 287  332  311 | 0.379 |
| **Fall**  No  Yes | 275  26 | 285  410 | 0.379 |
| **Pain**  No  Yes | 257  44 | 289  298 | 0.979 |

OS indicates overall survival; ESA, erythropoiesis stimulating agents; TSA, thrombopoiesis stimulating agents; ICT, iron chelation therapy; LD-Ara-C, low-dose cytarabine; CTX, chemotherapy; CR, complete response; allo-SCT, allogeneic stem cell transplantation; GIT, gastro-intestinal tract; AE, adverse event; AZA, azacitidine;

^1^Log-Rank (Mantel-Cox);

^2^Target dose = 75 mg/m^2^ x 7

^3^i.e. WHO-AML patient treated with azacitidine first line AND with 20-30% bone marrow blasts.
