# Supplementary material for: Azacitidine in 302 patients with WHO-defined acute myeloid leukemia: results from the Austrian Azacitidine Registry of the AGMT-Study Group
Source: Ann Hematol. 2014 Jun 21;93(11):1825–38. doi: 10.1007/s00277-014-2126-9 (PMC4176957; doi:10.1007/s00277-014-2126-9)
Supplement: Supplementary file 5 — (DOCX 36 kb) [file 277_2014_2126_MOESM5_ESM.docx]

**Supplemental Table 5. Factors that significantly affected overall survival**

|  | | | **Univariate Analysis** | | | **Multivariate Analysis** | | | | |
| --- | --- | --- | --- | --- | --- | --- | --- | --- | --- | --- |
| **Variable** | **n** | **Median OS, days** | ***p*-value**^1^ | **HR** | **95% CI** | ***p*-value**^1^ | **HR** | | **95% CI** | |
| **Baseline factors** | | | | | | | | | | |
| **PB-blasts**  0%  > 0% | 101  186 | 404  232 | **0.002 | 1.529 | 1.159-2.017 | *p*=0.122  (did not meet 0.05 criterium for model entry); | | | | |
| **PLT-TD prior to AZA**  No  Yes | 188  113 | 311  281 | *0.037 | 1.317 | 1.015-1.710 | *p*=0.395  (did not meet 0.05 criterium for model entry); | | | | |
| **LDH**  ≤ 225 U/l  > 225U/l | 134  163 | 390  220 | **0.001 | 1.536 | 1.177-2.003 | *0.025 | 1.349 | | 1.038-1.754 | |
| **ECOG performance score**  < 2  ≥ 2 | 174  59 | 325  257 | **0.009 | 1.490 | 1.104-2.009 | *0.025 | 1.429 | | 1.046-1.952 | |
| **Number of comorbidities**  ≤ 3  > 3 | 205  34 | 294  168 | *0.023 | 1.582 | 1.062-2.359 | **<0.001 | 1.740 | | 1.304-2.322 | |
| **HCT-CI**  Low risk  Intermediate risk  High risk | 93  117  91 | 390  289  262 | *0.046 | 1.224 | 1.041-1.440 | *p*=0.157  (did not meet 0.05 criterium for model entry); | | | | |
| **IPSS cytogenetic risk score**  Good  Intermediate  Poor | 161  55  52 | 382  230  155 | **0.001 | 1.373 | 1.161-1.624 | *p*=0.189  (did not meet 0.05 criterium for model entry); | | | | |
| **MRC cytogenetic risk group**  Good  Intermediate  Poor | 11  201  56 | 246  319  155 | *0.024 | 1.393 | 1.030-1.885 | *p*=0.548  (did not meet 0.05 criterium for model entry); | | | | |
| **Adverse karyotype (-7, -7q, abn(3q), complex)**  No  Yes | 213  53 | 319  155 | **0.001 | 1.742 | 1.252-2.423 | *p*=0.811  (did not meet 0.05 criterium for model entry); | | | | |
| **Monsomal karyotype**  No  Yes | 236  32 | 298  123 | **0.001 | 1.947 | 1.310-2.896 | **0.002 | 1.293 | | 1.096-1.526 | |
| **APSS (azacitidine prognostic scoring system)**  Low  Intermediate  High | 38  188  29 | 400  273  123 | **0.004 | 1.551 | 1.172-2.054 | *p*=0.289  (did not meet 0.05 criterium for model entry); | | | | |
| **Prior ‘imids’ (thalidomide or lenalidomide)**  No  Yes | 286  15 | 294  138 | *0.021 | 1.968 | 1.094-3.540 | *p*=0.492  (did not meet 0.05 criterium for model entry); | | | | |
| **Pretreatment with clinical trial medications/others**  No  Yes | 286  15 | 294  126 | *0.022 | 1.913 | 1.088-3.362 | *p*=0.136  (did not meet 0.05 criterium for model entry); | | | | |
| **Pretreatment with chemotherapy (CTX)**  No CTX  ≥1 prior line of CTX | 176  125 | 329  232 | **0.010 | 1.402 | 1.082-1.817 | *p*=0.365  (did not meet 0.05 criterium for model entry); | | | | |
| **AZA 1st line**^2^  No  Yes | 163  138 | 228  390 | **<0.001 | 0.599 | 0.461-0.779 | *0.015 | 1.103 | | 1.019-1.194 | |
| **FDA-label (AZA 1st line AND <=30% BM blasts)**  No (off-label)  Yes (on-label) | 252  49 | 275  416 | *0.011 | 0.617 | 0.424-0.899 | *p*=0.122  (did not meet 0.05 criterium for model entry); | | | | |
| **Time-dependent factors – Response** | | | | | | | | | | |
| **Hematologic improvement – IWG**^3^  No  Yes | 181  120 | 137  490 | **<0.001 | 0.804 | 0.751-0.860 | *0.042 | 0.675 | | 0.462-0.986 | |
| **RBC-TI IWG**^3^  No  Yes | 47  68 | 287  521 | **<0.001 | 0.328 | 0.210-0.512 | *ND* due to redundancy of the variable with ‘hematologic improvement’; | | | | |
| **PLT-TI IWG**^3^  No  Yes | 29  47 | 267  573 | **0.005 | 0.487 | 0.293-0.811 | *ND* due to redundancy of the variable with ‘hematologic improvement’; | | | | |
| **Best marrow response - ITT**  No (mSD, no response, PD)  Yes (CR, mCR, PR) | 213  88 | 175  594 | **<0.001 | 0.305 | 0.224-0.415 | *p*=0.642  (did not meet 0.05 criterium for model entry); | | | | |
| **Overall response**^4^ **- ITT**  No  Yes | 157  144 | 114  489 | **<0.001 | 0.307 | 0.235-0.401 | *ND* due to redundancy of the variable with ‘Best marrow response’ and ‘Hematologic improval’; | | | | |
| **Overall response**  mCR/CR/PR with HI  mSD with HI  mCR/CR/PR without HI  HI only  mSD without HI  No response/PD | 64  44  24  12  22  135 | 623  574  456  294  246  96 | **<0.001 | 1.191 | 1.150-1.234 | *ND* due to redundancy of the variable with ‘Best marrow response’ and ‘Hematologic improval’; | | | | |
| **Response deepening**  No response  1^st^ response = best response  1^st^ response < best response | 157  99  45 | 114  382  650 | **<0.001 | 0.440 | 0.363-0.535 | **<0.001 | 0.398 | | 0.324-0.490 | |
| **Time-dependent factors – Toxicity and adverse events** | | | | | | | | | | |
| **Infectious complications**  None/Grade 1-2  Grade 3-4 | 202  99 | 368  230 | **<0.001 | 1.558 | 1.195-2.032 | **<0.001 | 1.772 | | 1.323-2.374 | |
| **Fatigue Grade 3**  No  Yes | 264  37 | 315  121 | **<0.001 | 2.123 | 1.471-3.064 | *p*=0.053  (did not meet 0.05 criterium for model entry); | | | | |
| **Gastro-intenstinal-Toxicity**  None  Grade 1–2  Grade 3-4 | 234  65  2 | 267  414  14 | **0.001 | 0.681 | 0.496-0.934 | *p*=0.827  (did not meet 0.05 criterium for model entry); | | | | |
| **Hematologic toxicity Grade 3-4**  No  Yes | 157  144 | 246  382 | **<0.001 | 0.631 | 0.486-0.819 | **0.003 | | 0.647 | | 0.486-0.861 |
| **Grade 3-4 adverse events attributable to AZA**  No  Yes/Unknown | 63  54 | 190  456 | **<0.001 | 0.488 | 0.327-0.728 | *p*=0.280  (did not meet 0.05 criterium for model entry); | | | | |
| **Injection site reaction**  None  Painless ulcer, erythema, mild soreness  Pain, swelling, inflammation, phlebitis | 243  44  12 | 92  253  390 | *0.020 | 0.637 | 0.485-0.836 | *p*=0.144  (did not meet 0.05 criterium for model entry); | | | | |
| **Dose reduction due to adverse events**  No  Yes | 255  46 | 273  492 | **0.004 | 0.591 | 0.413-0.846 | *p*=0.051  (did not meet 0.05 criterium for model entry); | | | | |
| **AZA pause due to adverse events**  No  Yes | 227  75 | 262  437 | **0.001 | 0.604 | 0.446-0.817 | **<0.001 | | 0.543 | | 0.393-0.749 |

mo determines months; HR, hazard ratio; CI, confidence interval; PB, peripheral blood; PLT-TD, platelet transfusion dependence; ITT, intention to treat analysis, CR, complete response; mCR, marrow CR; PR, partial response; mSD, marrow stable disease; PD, progressive disease; OR, overall response; ND, not done;

^1^Log-Rank (Mantel-Cox);

^2^Defined as patients without prior disease modifying treatment (i.e. growth-factors and iron chlation were allowed).

^3^According to 2006 modified IWG criteria (i.e. those patients who received ≥ 2 cycles of azacitidine and remained transfusion independent for ≥ 8 weeks)

^4^Definded as CR, mCR, PR and/or HI
