# Supplementary material for: Azacitidine in 302 patients with WHO-defined acute myeloid leukemia: results from the Austrian Azacitidine Registry of the AGMT-Study Group
Source: Ann Hematol. 2014 Jun 21;93(11):1825–38. doi: 10.1007/s00277-014-2126-9 (PMC4176957; doi:10.1007/s00277-014-2126-9)
Supplement: Supplementary file 6 — (DOCX 37 kb) [file 277_2014_2126_MOESM6_ESM.docx]

**Supplemental Table 6. Comparison of characteristics of n=155 and n=302 cohorts**

|  | **n=302** | **n=155** | ***p*-Value** |
| --- | --- | --- | --- |
| **Comparison of baseline characteristics** | | | |
| **Median age, years** | 73 | 73 | 1.0 |
| **Gender, male, %** | 58 | 59 | 0.837 |
| **WHO Diagnosis, %**^1,2^  t-AML  AML-RCA^3^/gene mutations  AML-MRF  AML-NOS | 8  18  54  20 | 10  10  57  23 | 0.424 |
| **Peripheral blood blasts, %**  No data  0%  > 0% | 5  33  62 | 5  32  63 | 0.99 |
| **Bone marrow blasts, %**  <20%^5^  20–30%  >30% (off label use) | 17  26  57 | 17  20  63 | 0.582 |
| **WBC count, n (%)**  ≥ 10 G/l | 21 | 21 | 1.0 |
| **Transfusion dependence, n (%)**  RBC-TD  PLT-TD  RBC-TD + PLT-TD | 57  36  34 | 63  39  36 | 0.99 |
| **IPSS cytogenetic risk, n (%)**  Not evaluable  Good  Intermediate  Poor | 11  54  18  17 | 7  59  16  17 | 0.747 |
| **MRC cytogenetic risk, n (%)**  Not evaluable  Good  Intermediate  High | 11  3  66  19 | 7  2  74  17 | 0.648 |
| **Specific chromosomal aberrations, %**  Complex karyotype  -5q  -7  -7q  +8  -Y | 10  11  8  6  9  3 | 8  10  7  7  7  3 | 0.996 |
| **Comorbidities**^2^**, %**  Thromboembolic  Renal insufficiency  Liver disease  Diabetes mellitus  Coronary artery disease  Pulmonary  Prior malignanicies | 9  18  10  17  22  8  22 | 14  19  13  17  22  10  23 | 0.981 |
| **Number of comorbidities, %**  0-1  2-3  >3 | 47  34  18 | 43  39  18 | 0.773 |
| **ECOG ≥2, %** | 24 | 26 | 0.639 |
| **HCT-CI, %**  Low risk  Int. risk  High risk | 31  39  30 | 30  30  32 | 0.63 |
| **Treatment prior to AZA**^2^**, %**  None  Erythropoietin stimulating agents  G-CSF  Thrombopoietin stimulating agents  Iron chelation therapy  Thalidomide  Lenalidomide  ATG, CyA  Low-dose cytarabine  Intensive chemotherapy for MDS/AML  Others | 38  8  11  1  4  2  4  1  4  41  14 | 41  10  12  1  3  2  4  0  7  39  10 | 0.999 |
| **Reason for treatment, %**  First line treatment^8^  Bridging to allo-SCT  Maintenance after CR to CTX  No CR to conventional chemotherapy/allo-SCT  No CR to other disease modifying treatment | 38  3  5  32  22 | 41  3  4  32  21 | 0.993 |
| **Reason for AZA stop, %**  Disease relapse/progression  No response  Toxicity/infectious complications  Death  Allogeneic stem cell transplantation  Patient’s wish  Others  No data | 29  11  7  30  1  7  14  0 | 32  13  10  25  3  6  10  1 | 0.776 |
| **≤2 cycles of AZA, %**  Death within 1 month of AZA stop  Death within 2 months of AZA stop | 34  40  56 | 31  48  54 | 0.446 |
| **Comparison of treatment modalities** | | | |
| **Concomitant treatment, %**  Erythropoietin stimulating agents  G-CSF  Iron chelation therapy  Hospitalization for AZA treatment | 2  19  2  47 | 3  21  3  44 | 0.846  0.572  0.703  0.542 |
| **AZA application route**  s.c.  i.v.  i.v. + s.c. | 85  10  5 | 88  8  4 | 0.825 |
| **FDA target dose**  < Target dose  = Target dose  > Target dose | 38  59  2 | 45  53  2 | 0.635 |
| **AZA schedule all patients, %**  AZA 1-5  AZA 1-7  AZA 5-2-2  AZA others | 16  53  24  7 | 16  57  22  5 | 0.904 |
| **AZA schedule responders, %**  AZA 1-5  AZA 1-7  AZA 5-2-2  AZA others | 16  49  29  7 | 14  52  30  4 | 0.788 |
| **Comparison of adverse events** | | | |
| **Grade 3-4 hematologic toxicity, %**  Thrombocytopenia  Neutropenia  Anaemia | 46  30  33  27 | 45  25  32  21 | 0.833 |
| **Grade 3-4 infectious complications, %**  Febrile neutropenia | 33  18 | 29  18 | 1 |
| **Grade 3-4 non-hematologic toxicity, %**  Liver  Kidney  Heart/blood pressure^‡^  Thromboembolic  Neurologic  Gastrointestinal  Injection site reaction | 2  3  11  1  0  1  1 | 0  0  9  1  1  0  0 | 0.366 |
| **Fatigue limiting self care, %** | 12 | 10 | 0.522 |
| **Severe pain, %** | 3 | 2 | 0.529 |
| **Novel solid tumor, %** | 1 | 2 | 0.379 |
| **Emergency surgery, %** | 4 | 5 | 0.619 |
| **Fall with fracture or hemorrhage, %** | 7 | 9 | 0.447 |
| **Adverse events (AE) attributable to AZA, %**  No  Yes  Unknown | 63  24  13 | 63  32  5 | 0.095 |
| **Grade 3-4 AE attributable to AZA, %**  No  Yes  Unknown | 64  16  20 | 70  20  10 | 0.132 |
| **Consequence of AE, %**  None  Treatment  Hospitalisation  Life threatening/ICU  Death | 35  33  23  1  9 | 35  35  22  1  6 | 0.956 |
| **Consequence for AZA treatment**  None  Dose reduction  Treatment pause  Termination of AZA treatment  Prolongation of cycle >28 days | 65  5  11  11  7 | 69  6  14  9  1 | 0.261 |
| **Comparison of response** | | | |
| **Transfusion independence, % ITT, [%IWG]**  PLT-TI  RBC-TI | 42 [62  39 [60] | 40 [56]  36 [51] | 0.920 [0.818] |
| **Hematologic improvement, % ITT, [%IWG]**  HI-any  HI-PLT  HI-n.Gr.  HI-ery  No HI | 39 [60]  29 [44]  15 [23]  30 [45]  61 [41] | 32 [46]  21 [30]  14 [22]  23 [33]  68 [54] | 0.637 [0.184] |
| **Marrow response, % ITT, [%evaluated]**  Yes  CR  mCR  PR  No  mSD  primary PD | 30 [65]  13 [28]  4 [9]  13 [28]  11 [24]  5 [10] | 34 [69]  10 [20]  3 [7]  21 [43]  12 [25]  3 [5] | 0.537 [0.206]  0.474 [0.23] |
| **Overall response, % ITT, [%IWG]**  Yes  No | 48 [72]  52 [28] | 45 [65]  55 [35] | 0.671 [0.287] |
| **1^st^ response by, %**  cycle 3  cycle 4  cycle 5 | 58  78  87 | 44  77  87 | 0.495 |
| **Best response by, %**  cycle 5  cycle 6  cycle 7 | 67  81  90 | 67  80  88 | 0.995 |
| **1^st^ response = best response, %** | 69 | 66 | 0.514 |
| **1^st^ response < best response, %** | 31 | 34 | 0.514 |
| **AZA schedule (responders), %**  predominantly 1-5  predominantly 1-7  predominantly 5-2-2  predominantly others | 15  50  28  7 | 14  52  30  4 | 0.811 |
| **AZA dose (responders), %**  <500 mg/cycle  500-600 mg/cycle  601-800 mg/cycle  >800 mg/cycle | 4  6  25  65 | 3  6  27  65 | 0.975 |
| **Median number of cycles responder** | 9 (1-37) | 9 (1-31) | 1 |
| **Median number of cycles non-responder** | 2 (1-28) | 2 (1-21) | 1 |
