# Supplementary material for: Azacitidine in 302 patients with WHO-defined acute myeloid leukemia: results from the Austrian Azacitidine Registry of the AGMT-Study Group
Source: Ann Hematol. 2014 Jun 21;93(11):1825–38. doi: 10.1007/s00277-014-2126-9 (PMC4176957; doi:10.1007/s00277-014-2126-9)
Supplement: Supplementary file 7 — (DOCX 24 kb) [file 277_2014_2126_MOESM7_ESM.docx]

**Supplemental Table 7. Number of AML diagnoses per year in Austria, and patient recruitment to the AAR**

|  | **Statistiks Austria^1^** | **Data from AML patients included in AAR** | | | **Data from Tumor Registry Salzburg^2^** | **Data from AML patients included in AAR** | |
| --- | --- | --- | --- | --- | --- | --- | --- |
|  | n AML diagnoses  in Austria^1,3^ | n AZA start  in AAR^4^ | n entered in eCRF  in Austria | n centers entering AML pts. in eCRF in AAR | n AML diagnoses  in Salzburg | n AZA start  in Salzburg^4^ | n entered in eCRF  in Salzburg^4^ |
| **2007** | 318 | 10 | n.a.^5^ | n.a.^5^ | 26 | 4 | n.a.^4^ |
| **2008** | 279 | 22 | n.a.^5^ | n.a.^5^ | 33 | 13 | n.a.^4^ |
| **2009** | 269 | 55 | 56 | 4 | 20 | 14 | 28 |
| **2010** | 316 | 59 | 48 | 10 | 26 | 15 | 16 |
| **2011** | 266 | 64 | 47 | 8 | 31 | 11 | 7 |
| **2012** | n.g. | 78 | 96 | 14 | 44 | 13 | 23 |
| **2013** | n.g. | 41 | 61 | 11 | 43 | 11 | 6 |
| **Total** | **1448** | **329** | **308** | **15** | **223** | **81** | **80** |

AAR indicates Austrian Azacitidine Registry; AZA, azacitidine; eCRF, electronic case report form; n.g., not given; n.a., not applicable;

^1^Data received from Statistiks Austria on 23.05.14

^2^patients aged >20 years

^3^Data received from Tumor Registry Salzburg on 26.05.14

^4^Please note, that the patients started on azacitidine in a respective year, are not necessarily those diagnosed in the same year; data cut-off 26.05.14;

^5^Patient documentation was started as of ethics committee approval, which was obtained 01.02.2009;

**Supplemental Table 7** shows the number of AML new diagnoses per year in Austria. In addition, the table shows the numbers of AAR-patients started on azacitidine per respective year, the number of centers including patients in the AAR per respective year, as well as the number of patients included in the AAR per respective year (note: as ethics committee approval was obtained 01.02.2009, data entry commenced as of this time-point). The respective numbers for the center of Salzburg only are also shown.
